# Expression of the OAS Gene Family Is Highly Modulated in Subjects Affected by Juvenile Dermatomyositis, Resembling an Immune Response to a dsRNA Virus Infection

**DOI:** 10.3390/ijms19092786

**Published:** 2018-09-17

**Authors:** Giuseppe Musumeci, Paola Castrogiovanni, Ignazio Barbagallo, Daniele Tibullo, Cristina Sanfilippo, Giuseppe Nunnari, Giovanni Francesco Pellicanò, Piero Pavone, Rosario Caltabiano, Roberto Di Marco, Rosa Imbesi, Michelino Di Rosa

**Affiliations:** 1 Department of Biomedical and Biotechnological Sciences, Human Anatomy and Histology Section, School of Medicine, University of Catania, 95131 Catania, Italy; g.musumeci@unict.it (G.M.); pacastro@unict.it (P.C.); cri.sanfilippo@gmail.com (C.S.); roimbesi@unict.it (R.I.); 2 Department of Drug Sciences, University of Catania, 95100 Catania, Italy; ignazio.barbagallo@unict.com; 3 Department of Biomedical and Biotechnological Sciences, University of Catania, 95131 Catania, Italy; danieletibullo@gmail.it; 4 IRCCS Centro Neurolesi Bonino Pulejo, Stada Statale 113, C.da Casazza, 98124 Messina, Italy; 5 Department of Clinical and Experimental Medicine, Unit of Infectious Diseases, University of Messina, 98122 Messina, Italy; gnunari@hotmail.it (G.N.); pellicano@unime.com (G.F.P.); 6 Division of Pediatrics and Pediatric Emergency, University-Hospital “Policlinico-Vittorio Emanuele”, University of Catania, 95131 Catania, Italy; ppavone@unict.com; 7 Department “G.F. Ingrassia”, Section of Pathologic Anatomy, University of Catania, 95131 Catania, Italy; rosario.caltabiano@unict.com; 8 Department of Medicine and Health Sciences “V. Tiberio”, University of Molise, 86100 Campobasso, Italy; roberto.dimarco@unimol.it

**Keywords:** OAS1, OAS2, OAS3, OASL, muscles biopsies, dsRNA, JDM

## Abstract

Background: Juvenile dermatomyositis (JDM) is a systemic, autoimmune, interferon (IFN)-mediated inflammatory muscle disorder that affects children younger than 18 years of age. JDM primarily affects the skin and the skeletal muscles. Interestingly, the role of viral infections has been hypothesized. Mammalian 2′-5′-oligoadenylate synthetase (OAS) genes have been thoroughly characterized as components of the IFN-induced antiviral system, and they are connected to several innate immune-activated diseases. The main purpose of the paper is to define the potential interrelationship between the OAS gene family network and the molecular events that characterize JDM along with double-stranded RNA (dsRNA) molecular pathways. Methods: We analyzed three microarray datasets obtained from the NCBI in order to verify the expression levels of the OAS gene family network in muscle biopsies (MBx) of JDM patients compared to healthy controls. Furthermore, From GSE51392, we decided to select significant gene expression profiles of primary nasal and bronchial epithelial cells isolated from healthy subjects and treated with polyinosinic-polycytidylic acid (poly(I:C)), a synthetic analog of double-stranded RNA (dsRNA), a molecular pattern associated with viral infection. Results: The analysis showed that all OAS genes were modulated in JDM muscle biopsies. Furthermore, 99% of OASs gene family networks were significantly upregulated. Of importance, 39.9% of modulated genes in JDM overlapped with those of primary epithelial cells treated with poly(I:C). Moreover, the microarray analysis showed that the double-stranded dsRNA virus gene network was highly expressed. In addition, we showed that the innate/adaptive immunity markers were significantly expressed in JDM muscles biopsies. and that their levels were positively correlated to OAS gene family expression. Conclusion: OAS gene expression is extremely modulated in JDM as well as in the dsRNA viral gene network. These data lead us to speculate on the potential involvement of a viral infection as a trigger moment for this systemic autoimmune disease. Further in vitro and translational studies are needed to verify this hypothesis in order to strategically plan treatment interventions.

## 1. Introduction

Juvenile dermatomyositis (JDM) is a serious pediatric systemic autoimmune disease primarily affecting the proximal muscles and skin. JDM cutaneous manifestations can be difficult to treat and may progress to ulcerative diseases or subcutaneous calcifications [1]. The incidence is approximately three times higher in girls than boys [2,3]. The mean age of onset is seven years old [3]. Extra-muscular manifestations include arthritis, myocarditis, dilated cardiomyopathy, and vasculopathy [4].

Previous studies indicate that innate immunity plays a pivotal role in the pathogenesis of JDM [5]. Mature dendritic cells (DCs) and macrophages have been identified in inflamed muscles and in the skin from patients with JDM [6,7]. These cell types are capable of driving type I interferon (IFN) responses [8]. JDM is characterized by the presence of inflammatory infiltrates especially in the perimysium, as well as ischemia and pathognomonic perifascicular muscle fiber atrophy [9]. Adaptive and innate immune mechanisms involving IFN-associated molecules appear to mediate endothelial tubule-reticular formations and peri-fascicular atrophy [10,11].

The oligoadenylate synthase (OAS) proteins are double-stranded (ds) RNA-activated enzymes which are induced by IFNs. The OAS family consists of four members, OAS1, OAS2, OAS3, and OAS-like protein (OASL) [12]. These proteins sense exogenous nucleic acid and initiate antiviral pathways. The OAS enzyme develops a 2′-5′-linked oligoadenylate (2-5A) activating endoribonuclease, RNase L, which degrades cellular and viral RNA, and activates cytoplasmic receptors, such as RIG-I and MDA-5. Although the antiviral activity of the OAS family and 2-5A-RNase L is amply described [13,14], it has been shown that the RNase L-dependent activity does not always act in the antiviral functions mediated by the OAS family [15,16]. All of this could indicate that the OAS family proteins may be involved in pathways regulating viral infections independently by the involvement of RNase L activation. OAS expression has been strongly related to autoimmune diseases and chronic infections including, SSc, SLE, RA, and MS [17,18,19,20]. Recently, it was shown that OAS1 is upregulated and OASL downregulated in systemic lupus erythematosus compared to healthy individuals [20]. 

In this paper, we explored the possible role of OAS genes antiviral response in JDM. The analysis of the OAS gene family and the genes related to it suggests that dsRNA viral infections could be involved in the pathogenesis of this disease. 

## 2. Results

### 2.1. High Expression Levels of the OAS Gene Family in MBx of JDM Patients

The GSE3307 contained 13 groups of datasets of several muscle diseases. The available groups were: normal human skeletal muscle (*n* = 20), acute quadriplegic myopathy (AQM; critical care myopathy) (*n* = 3), JDM (*n* = 21), amyotrophic lateral sclerosis (ALS) (*n* = 9), spastic paraplegia (SPG4; spastin) (*n* = 4), fascioscapulohumeral muscular dystrophy (FSHD), Emery Dreifuss muscular dystrophy (both X-linked recessive emerin forms; autosomal-dominant Lamin A/C form (LMNA) (*n* = 22), Becker muscular dystrophy (partial loss of dystrophin) (*n* = 5), Duchenne muscular dystrophy (complete loss of dystrophin) (*n* = 10), Calpain 3 (LGMD2A) (*n* = 9), dysferlin (LGMD2B) (*n* = 10), and fukutin related protein FKRP (glycosylation defect; homozygous for a missense mutation) (*n* = 7). 

We hypothesized that a possible explanation that the JDM etiogenesis could be a viral infection, as previously predicted [21]. In order to answer this question, we decided to overlap the genes significantly modulated in JDM MBx compared to healthy donors and the key genes involved in a hypothetical viral infection. 

**Figure 1:**
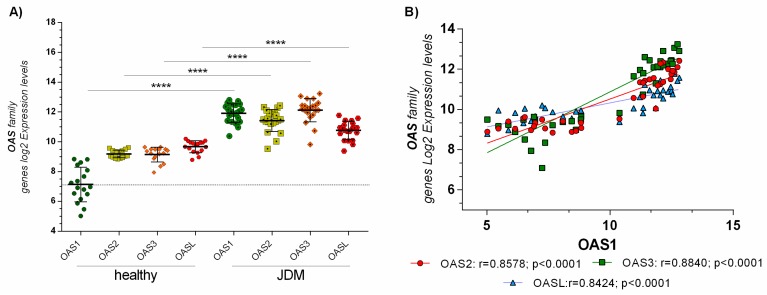
(**A**) Skeletal muscle biopsies of JDM patients express significantly higher levels of OAS genes family: OAS1, OAS2, OAS3, and OASL expression levels were significantly upregulated (*p* < 0.00001) in MBx of JDM patients compared to healthy controls. (**B**) Furthermore, OAS gene family expression was positively correlated with each other in JDM MBx. Dataset accession number GSE3307. Data are expressed as log_2_ intensity expression levels and presented as vertical scatter dot plots. *p* values < 0.05 were considered to be statistically significant (* *p* < 0.05; ** *p* < 0.005; *** *p* < 0.0005; **** *p* < 0.00005).

From a preliminary analysis, we showed that the OAS genes family were highly modulated (Supplementary Table S1). These genes play a crucial role during an infection mediated by a dsRNA virus. We found that OAS1 (*p* < 0.001), OAS2 (*p* < 0.001), OAS3 (*p* < 0.001), and OASL (*p* < 0.001) were highly expressed in JDM MBx compared to healthy controls (Figure 1A). Also, OAS genes family expression was positively correlated with each other in JDM MBx. No correlation was observed in healthy controls (Figure 1B).

No modulation was observed in the other muscle diseases (data not shown). Furthermore, none of the expression values of OAS1 and OAS3 genes in the healthy group fell in the expression range of the JDM group (Figure 1A). 

To validate these results, we decided to perform a new analysis of two more different microarray datasets (GSE11971 and GSE48280) composed by the MBx of healthy donor and children affected by JDM. The analysis confirmed the results obtained for GSE3307 (Supplementary Figure S1A,B and Table S1).

### 2.2. The Network of OAS Genes and Their Possible Roles in JDM

In order to verify if the OASs genes could be involved in the JDM pathogenesis, we performed a gene network analysis. For this reason, we interrogated the GIANT and STRING online software.

**Figure 2:**
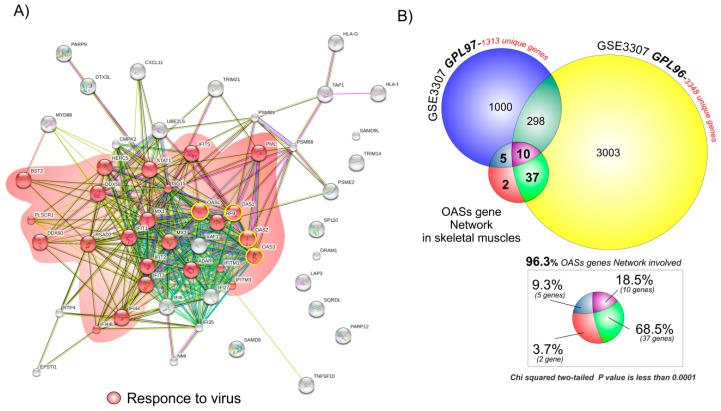
96.3% of the OAS gene network is significantly upregulated in MBx of JDM patients: (**A**) We built a STRING network of 54 OASs genes determined by GIANT analysis. The genes shown in red belong to a response to a virus. (**B**) Venn diagram tools were drawn using the web-based utility Venn Diagram Generator, and the graphical representation was realized with CorelDraw ×6. Weighted Gene Networks were built with the STRING online tool based on the results obtained by the GIANT software, and subsequently graphically modified with CorelDraw ×6. The 54 genes obtained by the GIANT software analysis were overlapped to the significantly upregulated genes in MBx of JDM patients vs. healthy controls. Among these 54 genes, 52 (96.3%) were upregulated in both the JDM and the healthy group (*p* < 0.0001), and two (3.7%) unequivocally belonged to the OAS network.

For the GIANT online tool, we set the tissue menu as skeletal muscles, the network filter with a minimum relationship confidence of 0.18, and the maximum number of genes as 50. The list of 54 genes (including the four OASs genes) obtained was loaded into the multiple sequences search set of the STRING software in order to obtain a graphical network representation (Figure 2A). 

STRING analysis showed a large number of genes belonging to a specific biological process (response to the virus). These results were confirmed by a gene ontology analysis performed with FunRich online tools (Supplementary Figure S2).

The 54 genes obtained by the GIANT analysis were compared to the significantly upregulated genes in GSE3307 (3348 for GPL96 and 1313 for GPL97) (Figure 2B). Among these 54 genes, 52 (96.3%) were common between the JDM and the healthy control upregulated genes (*p* < 0.0001) (Figure 2B). No significantly common genes to the OASs gene network were observed among the list of downregulated genes (data not shown). These results were confirmed when we compared the significantly upregulated genes to the OASs gene network of all three microarray datasets selected for this study (GSE3307, GSE11971 and GSE48280). We showed that 40 genes (74%) were common to the JDM and healthy control upregulated genes (*p* < 0.0001) (Supplementary Figure S3 and Table S1). 

All these results confirmed a potential role played by the OAS gene family network in the muscles of patients with JDM.

### 2.3. dsRNA Genes Expression Profiles Overlap with Transcriptome of JDM MBx

**Figure 3:**
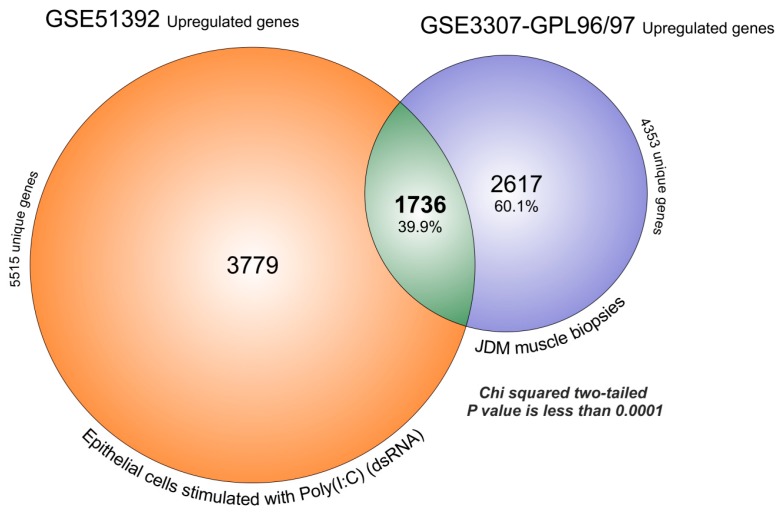
MBx of JDM transcriptome overlaps for 39.88% with significantly upregulated genes expressed in primary epithelial cells treated with polyinosinic-polycytidylic acid poly(I:C). The FDR analysis obtained by GEO2R, produced for GSE51392, 5515 significantly upregulated genes and for GSE3307, 4353 genes. The overlaps produced 1736 genes that were significantly upregulated in common (39.9%), and 2617 (60.1%) characteristics exclusive to JDM (chi-square two-tailed with a *p* value < 0.0001).

In the previous sections, we have shown that OAS pathway is associated with the response to viral infections (String and FunRich analysis). Starting from this result, we decided to overlap the transcriptome of GSE51392 composed by primary epithelial cells treated with polyinosinic-polycytidylic acid (poly(I:C)) (20 µg/mL for 24 h) and GSE3307 microarray dataset, based on JDM MBx. The FDR analysis obtained by GEO2R, produced for GSE51392 a large number of significantly upregulated genes (5515 genes) (Supplementary Table S1). The overlapped with GSE3307, producing 1736 genes that were significantly upregulated in both (39.9%), and 2617 (60.1%) that were exclusive for JDM (chi-square two-tailed with a *p*-value <0.0001) (Figure 3).

**Figure 4:**
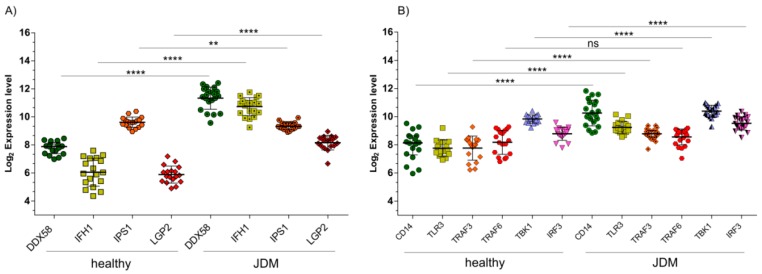
Endosomal gene pathways activated during dsRNA virus infections. (**A**) RIG-I (DDX58), MDA5 (IFIH1), and LGP2 expression levels are significantly upregulated (*p* < 0.00001) in MBx of JDM patients compared to healthy controls. On the contrary, IPS1 was significantly downregulated (*p* < 0.001). (**B**) The GSE3307 analysis reveals a significant upregulation of endosomal genes (CD14, TLR3, TRAF3, TBK1, and IRF3) activated during dsRNA virus infection in MBx of JDM patients vs. healthy controls. No significant modulation was observed for TRAF6. Data are expressed as log2 intensity expression levels, and presented as vertical scatter dot plots. *p* values < 0.05 were considered to be statistically significant (* *p* < 0.05; ** *p* < 0.005; *** *p* < 0.0005; **** *p* < 0.00005).

Inside the list of 1736 significantly upregulated genes that overlapped between GSE51392 and GSE3307, the OAS genes family was still present. Also, the dsRNA genes belonging to the transduction pathway, such as RIG-I (also known DDX58), IFIH1, IPS1 (also known MAVS), LGP2 (also known DHX58), CD14, TLR3, TBK1, and IRF, were part of those genes that were significantly modulated in common, in the two GSE (Figure 4) (Supplementary Figure S4 and Table S1). The expression levels of TRAF6 were an exception. A possible explanation for this event could be the downregulation of IPS-1 levels found in our analysis. In fact, it was largely demonstrated that RIG-like helicases signaling has been proposed to bifurcate at IPS-1 in two pathways, the TRAF3-dependent IRF activation pathway, and the TRAF6-dependent NF-κB activation pathway. The IPS-1 block could result in the downstream inhibition of TRAF6 [22]. With regard to TRAF3 upregulation, the signal divergence, and its regulation by TLR3 could explain its modulation.

Extracellular dsRNA or intracellular dsRNA activate different signaling pathways: TLR3 and CD14 (endosome pathways), or DDX58 or IFIH1 (Cytoplasmic pathways), respectively [23]. The microarray dataset analysis showed a significant upregulation of DDX58 (*p* < 0.0001), IFIH1 (*p* < 0.0001) (Figure 4A), and also CD14 (*p* < 0.0001) and TLR3 (*p* < 0.0001) (Figure 4B), in MBx of JDM patients compared to healthy controls. When TLR3 recognizes dsRNA, it dimerizes, binds to CD14, and activates the signaling complex assembled by TRIF, TRAF3, TBK1, and IRF3. Moreover, we found that TRIF (*p* < 0.01) was significantly downregulated (Supplementary Table S1), whereas TRAF3 (*p* < 0.001), TBK1 (*p* < 0.001), and IRF3 (*p* < 0.001) were significantly upregulated in JDM vs. healthy controls (Figure 4B). As for the cytoplasmic RNA helicases DDX58 and IFH1, these proteins recognize dsRNA or 5′-triphosphorylated single-stranded (ss)RNA and use the mitochondrial membrane-bound protein IPS1 as the specific adaptor. We showed that DDX58 and IFH1 were significantly upregulated (Figure 4A) in JDM vs. healthy controls, while IPS1 was significantly downregulated (*p* < 0.001) (Figure 4A). Similar to TRIF, IPS1 activates the same transcription factors leading to the induction of similar genes. We found that IPS1 regulatory protein, LGP2, was significantly upregulated (*p* < 0.0001) in JDM vs. healthy controls (Figure 4A). This finding can partly explain the downregulation of IPS1. Overexpression of LGP2 was able to inhibit RIG-I-mediated antiviral signaling both in the presence and absence of viral ligands [24,25]. Furthermore, it was demonstrated that LGP2 can inhibit antiviral signaling independently of dsRNA or virus infection intermediates by engaging in a protein complex with IPS-1 [26].

**Figure 5:**
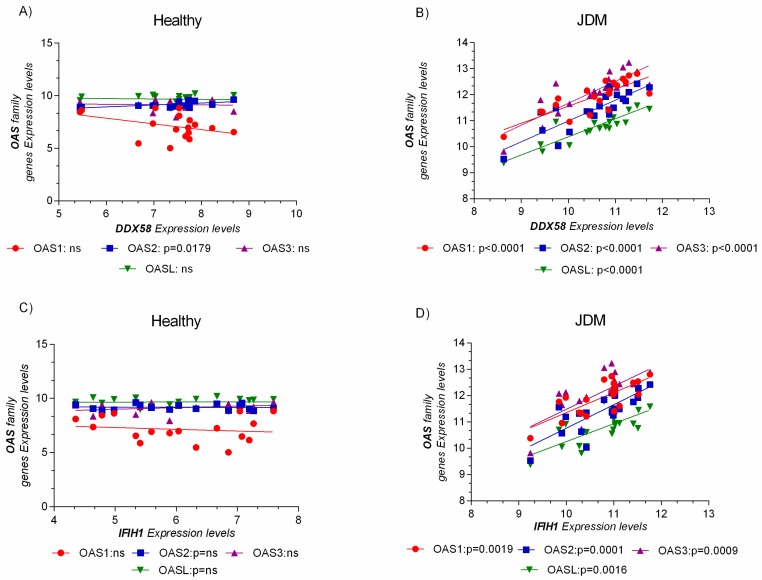
OAS gene expression levels were positively correlated to the pattern recognition receptor for virus’s infection. No correlation was observed in the healthy group (**A**,**C**). The expression level analysis of the OAS genes family and DDX58 (RIG-I, retinoic acid-inducible gene I), and IFIH1 (MDA5, melanoma differentiation-associated protein 5), reveals a positive correlation only in JDM MBxs (**B**,**D**). Data are expressed as log_2_ intensity expression levels and presented as dot plots. *p* values < 0.05 were considered to be statistically significant (* *p* < 0.05; ** *p* < 0.005; *** *p* < 0.0005; **** *p* < 0.00005).

Furthermore, the expression levels of OAS genes family were related to DDX58 (RIG-I, retinoic acid-inducible gene I) and IFIH1 (MDA5, melanoma differentiation-associated protein 5), the principal pattern recognition receptor (recognizing dsRNA), and a sensor for viral infection (Figure 5).

Similar results were obtained for GSE51392. Indeed, RIG-I (DDX58), IFIH1, OAS1, OAS2, OAS3, and OASL resulted significantly modulated and correlated to each other (Supplementary Figure S5 and Table S1).

### 2.4. The Innate Immunity Genes Markers Are Modulated in JDM Patients’ Biopsies

In order to verify the role played by the innate immunity and its correlation with OAS gene family expression in JDM patients’ biopsies, we performed an expression analysis of the main markers involved.

With regards to the innate immunity involvement in JDM, we analyzed cyclophilin A (PPIA, necrotic marker) [27], CD14, CD11b, CXCR4 (monocyte markers) [28], and CCL2 and CCL8 (monocyte infiltration markers) [29], in the datasets used for this study. A Z-score analysis of the tree datasets selected was performed in order to improve the significantly statistic results. We showed that all markers were significantly upregulated in JDM muscle biopsies compared to healthy subjects (Supplementary Figure S6).

The expression levels of all markers, excepting CD14, were significantly correlated with OAS genes family expression levels (Supplementary Table S1A). Furthermore, we analyzed the expression levels of IFNG [30] and its receptor (IFNGR1) [31] in order to verify whether these molecules play a role in JDM. The analysis showed that only IFNGR1 was significantly modulated (Supplementary Figure S7 and Table S1A).

The correlation analysis revealed that IFNGR1 was significantly correlated with OAS gene family expression. This result was probably due to the fact that the production of IFNG is a prerogative of T lymphocytes, and not of innate immunity [32,33]. Overall, these results showed that the OAS family genes were correlated with the monocyte genes markers and with the action of IFNG through the action of its receptor.

In order to present a complete framework of the situation on the innate immunity activity, we decided to analyze the expression levels of monocyte activation markers such as IL18 [34], CD163 [35], LAMP3 [36], and CHI3L1 [37]. We showed that the expression levels of all of the genes selected were significantly upregulated in JDM muscle biopsies, and its levels positively correlated with OAS1, OAS2, and OAS3 (an exception was CD163 and CHI3L1 vs. OAS1) (Supplementary Figure S8 and Table S1B) (for the details of results, see the supplementary section).

All these findings would seem to confirm the involvement of innate immunity in the biopsies of patients with JDM, and a possible correlation with the OAS family gene expression.

### 2.5. Adaptive Immunity Seems to Be Involved in JDM

In order to complete the immunological pathways potentially involved in JDM, we decided to analyze the involvement of adaptive immunity.

In recent years, the role played by Major Histocompatibility Complex MHC class I/II in JDM has already been studied [38,39,40]. In our analysis, we decided to measure the expression levels of MHC class I/II and the principal chemokines involved in the recruitment and activation of T and B lymphocytes. We showed that all MHC class I expression levels were more highly expressed than MHC class II [38] (Supplementary Figure S9).

Furthermore, the MHC class I expression levels were significantly correlated with OAS gene expression (Supplementary Table S1C). No correlation was observed between the OAS genes and MHC class II. These results would seem to confirm a mediated antiviral response to MHC class I and OAS.

With regard to the analysis of genes referable to recruitment and activation of T and B lymphocytes, we selected CCL19 [41], CCL3 [42], CCL4 [43], CCL5 [44], and CXCL13 [45]. The expression analysis of these chemokines in our datasets showed significant increases in JDM patients compared to healthy subjects (Supplementary Figure S10).

The correlation analysis between OAS gene expression levels and CCL19 showed significant results. With regard to CCL4, CCL5, and CXCL13, we observed a significant correlation with OAS1, OAS2, and OAS3. CCL3 was only positively correlated with OASL (Supplementary Table S1D).

These results could explain the possible recruitment and subsequent activation of adaptive immunity in the muscular biopsies of patients affected by JDM.

## 3. Discussion

In this manuscript, we analyzed three different microarray datasets of MBx from patients with JDM, and we showed that almost 98% of the OAS genes family network was significantly upregulated. Surprisingly, the microarray analysis showed that the dsRNA virus gene network was highly expressed in the MBx of JDM patients (Figure 5). These data were confirmed when we overlapped the transcriptome of primary epithelial cells treated with poly(I:C), a model mimicking viral infection, and the JDM MBx dataset. Also, the analysis of genes characterizing innate and adaptive immunity revealed their involvement in JDM, mediated by OAS family gene expression. Taken together, these results suggest the possible involvement of the dsRNA pathway in the pathogenesis of the disease.

It has been demonstrated that type 1 IFNs are linked to the pathogenesis of some forms of myositis [46], although the mechanisms remain still largely unknown. Moreover, TRIs (tubuloreticular inclusions), which are macromolecular structures that are visible in dermatomyositis muscle endothelial cells biopsies [47] were identified as markers of type 1 IFN pathway signaling. The OAS gene family consists of homologous enzymes that are encoded by IFN-stimulated genes [48]. In vitro, the synthesis of the OAS gene family requires cell activation by dsRNA or ssRNA, with little secondary structure [49]. OAS expression seems to be sufficient to protect cells against infection with picornaviruses, such as encephalomyocarditis virus EMCV or Mengo viruses [14,50,51]. Moreover, a recent study suggested an important role for OAS genes in the monocytes of Human Immunodeficiency Virus HIV-infected individuals [52].

Several papers have shown that excessive keratinocyte apoptosis can be responsible for the development of skin lesions in subjects with dermatomyositis [53]. One of the possible apoptotic mechanisms could be activated by OAS genes. In fact, it has been shown that the OAS/RNaseL pathway is a novel effector of BRCA1- and IFN-gamma mediated apoptosis [54].

Furthermore, it is important to highlight that innate immunity could play a predominant role in the progression of JDM, before the induction of adaptive immune responses [55]. One of the pathways activated by monocytes to restrain viral infections is OAS/RNase L. Moreover, myocyte necrosis, which is typically observed in inflammatory myopathies [56,57], could induce monocyte infiltration [55,56,58], the subsequent maturation and production of chemokines that are able to localize T lymphocytes [59] with consequent production of gamma IFN [59], and the activation of OAS genes. This data was partially confirmed during our analysis. 

In addition, we found upregulation of the genes produced during extracellular or intracellular dsRNA viral replication. Recently, several papers have documented the modulation of MDA5 in JDM muscle biopsies [60]. This molecule is like an intracellular pathogen sensor that is located in the cytosol, and belongs to the family of RIG-I-like receptors (RLRs), as well as RIG-I [61]. In physiological conditions, it binds long (>1000 bp) viral dsRNA without any end-specificity. Recently, MDA5 has been identified as a DM-specific autoantigen that appears to be targeted in patients with DM and mild or absent muscle inflammation, and with an increased risk of interstitial lung disease [62]. The messenger RNA (mRNA) increase we observed in MBx may represent a compensatory mechanism in response to MDA5 inactivation by autoantibodies. Moreover, the inactivation of MDA5 pathways could make JDM patients more susceptible to dsRNA virus infection.

The hypothesis that the onset of autoimmune rheumatic diseases could be triggered by virus infection is controversial and based on case reports [63,64,65,66,67]. Several molecular mechanisms have been proposed to explain the role of infectious agents on the induction of autoimmune diseases as a molecular mimic, priming of reactive T cells or superantigenic T cell activation. Structural homology between infectious and host components or self-antigens that are processed and presented by Antigen-Presenting Cell APC, leading to priming of self-reactive T cells or viral products, which cross-link the T cell receptor and the MHC molecule independent of specific antigens, represent potential mechanisms that are hidden behind the JDM onset.

This paper proposes to explain the role of innate and adaptive immunity in JDM through OAS gene family action. Our observations led us to think that a plausible explanation of JDM etiogenesis could be that monocytes that are locally attracted in the muscle of those that are affected by JDM could transform into activated macrophages by proceeding with the elimination of the causative etiological agent (hypothetical double-strand virus). Subsequently, the release of chemokines specific for T and B lymphocytes could determine the polarization and activation (IFNG) of T lymphocytes. Moreover, the cytotoxic activity of CD8 T lymphocytes mediated by the major histocompatibility complexes of class I expressed by myocytes could determine the propagation of the disease.

These results are not conclusive. Further studies are needed to investigate the role of the OAS gene family in the disease mechanism, and whether they contribute to muscle tissue damage in patients with JDM disorders. 

## 4. Materials and Methods

### 4.1. Microarray Selection

In this manuscript, we evaluate microarray datasets from the NCBI GEO (http://www.ncbi.nlm.nih.gov/geo/) (access on 18 January 2018) databank under accession numbers GSE3307 (GPL96/97), GSE11971, GSE48280, and GSE51392 in order to delineate a correlation between the OAS gene family network and the molecular events that characterize JDM.

From the GSE3307 microarray dataset, we sorted the data of normal MBx (*n* = 20) from volunteers participating in exercise physiology studies, and MBx from patients with JDM (*n* = 21 female and *n* = 4 male). Complete experimental details can be retrieved in the publication by Bakay et al. and Dadgar et al. [68,69].

To validate the results obtained with GSE3307, we also analyzed two more microarray datasets (GSE11971 and GSE48280). From the GSE11971 microarray dataset, we selected the data of MBx from 16 girls with JDM not receiving any nonsteroidal or immunosuppressive therapy, and four healthy age- and sex-matched controls. As for the GSE48280 microarray dataset, we analyzed the data of MBx from five patients with JDM (all female) and five controls obtained from individuals during hip replacement surgery. The complete experimental details of the two microarray datasets can be found in the publications by Chen et al. and Suárez-Calvet et al. [70,71].

From GSE51392, we decided to select significant gene expression profiles of primary nasal and bronchial epithelial cells isolated from healthy subjects and treated with Polyinosinic-polycytidylic acid (poly I:C) 20µg/mL for 24 hr. This molecule is a synthetic immunostimulant mimicking a double-stranded RNA (dsRNA) structure. The poly(I:C) cell treatment is able to activate antiviral receptors such as TLR3, RIG-I/MDA5, and PKR, with subsequent induction of multiple inflammatory pathways, such as NF-kB and IRF. For more details refer to the publication by Wagener AH et al. [72].

The Multi-Experiment Viewer (MeV) software was chosen to identify differentially expressed genes. In cases where multiple probes insisted on the same NCBI GeneID, we selected those with the highest variance. By restricting the threshold level of significance to *p* < 0.01, for the GPL96 Platform of GSE3307, we identified 3348 upregulated and 6480 downregulated genes in JDM MBx vs. healthy MBx; for the GPL97 Platform, we found 1313 upregulated and 4693 downregulated genes. In order to exclude the genes redundant in the two platforms GPL96/97, we overlapped the significant up- and downregulated genes obtained during the analysis. The number unique upregulated genes obtained was 4353, and the unique downregulated genes was 8018. With regard to GSE11971, we identified 5395 upregulated and 2942 downregulated genes, comparing JDM MBx vs. healthy MBx. For GSE48280, we identified 1511 upregulated and 1621 downregulated genes. The analysis of GSE51392 produced the following results: 5515 upregulated and 6146 downregulated genes (Supplementary Table S1).

In order to identify genes that are commonly modulated in OAS gene network and JDM MBx, Venn diagrams were drawn using the online utility Venn Diagram Generator (http://www.bioinformatics.lu) (access on 18 January 2018). Weighted Gene Networks were obtained for the commonly modulated genes using STRING software (http://string-db.org/) (access on 18 January 2018) and GIANT software (http://giant.princeton.edu/) (access on 18 January 2018). The STRING combined score was determined with data from Neighborhood in the Genome, Experimental and Biochemical Data Co-occurrence Across Genomes [73].

The OASs gene pathway was obtained from the GIANT database (http://giant.princeton.edu/) (access on 18 January 2018), setting the tissue menu to skeletal muscles, the network filter to a minimum relationship confidence of 0.18, and the maximum number of genes to 50.

GIANT leverages a tissue-specific gold standard to automatically up-weight datasets that are relevant to a tissue from a large evidence compendium of diverse cell-types and tissues. [74].

GeneMANIA (https://genemania.org/) (access on 18 January 2018) is an online tool that is able to provide a global view of genes interactions. This information is derived from published experimental evidence and bioinformatics analysis predictions. Using the available online graphical user interface, we set the filters to 0 related genes and no related attributes, in order to restrict the gene network. The results for each gene were obtained by linear regression [75].

### 4.2. Functional Enrichment Analysis

Biological pathway, biological process, expression site heatmap, and analysis of KEGG pathways were drawn with the FunRich tool (http://www.funrich.org) (access on 20 January 2018) against the human FunRich background database [76].

### 4.3. Statistical Analysis

For statistical analysis and graphical representation, we used the Prism 7 software (GraphPad Software, 7825 Fay Avenue, Suite 230, La Jolla, CA 92037 USA). The Shapiro–Wilk test was used as the normality test. Significant differences between groups were assessed using the Mann–Whitney U test, and the Kruskal–Wallis test was performed to compare data between all groups, followed by Dunn’s post hoc test. Correlation analyses were determined using Spearman’s ρ correlation [77]. All tests were two-sided (significant value *p* < 0.05). A Chi-square with a Yates correction analysis was performed by GraphPad Prism software for a 2 × 2 contingency table (significant value *p* < 0.05). The analysis of the microarray data by a Z-score transformation was used in order to allow for the comparison of microarray data independent of the original hybridization intensities. [78].

## 5. Conclusions

**Figure 6:**
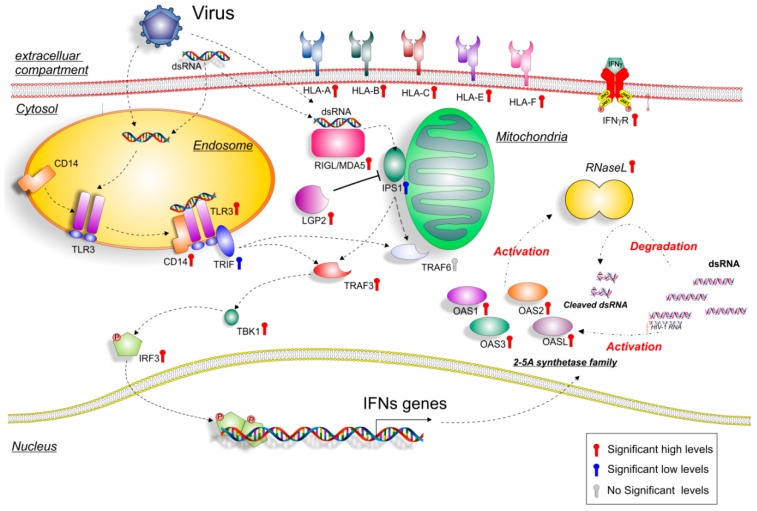
Hypothetical gene signaling pathways activated by dsRNA and viruses in JDM muscle biopsies. Extracellular or intracellular dsRNA produced during viral replication can activate different signaling pathways, such as endosome TLR3 or mitochondria RIG-I /MDA5. TLR3 dimerizes, binds to CD14 and activates TRIF, which activates in turn, TRAF3 and TBK1, and leads to IRF3 phosphorylation. The activated transcription factors translocate from the cytoplasm to the nucleus, bind to the cognate sites in the promoters of the target genes (OASs), and induce their transcription. As for the cytoplasmic RNA helicases RIG-I and MDA5, they recognize dsRNA and use the mitochondrial membrane-bound protein IPS1 as the specific adaptor. IPS1 functions like TRIF activate the same transcription factors, leading to the induction of similar genes. Colored thermometer indicates the significance values of genes (red: high significance level; blue: low significance level; grey: no significance level). For the genes depicted in the figure, refer to Supplementary Table S1.

In conclusion, our datasets analysis shows that OAS genes are upregulated in the muscle biopsies of JDM patients (Figure 6).

OASs gene expression could be induced by infiltrating monocytes, activation by dsRNA viruses, or by necrotic/apoptotic events in muscle myositis. Moreover, the activation of IFNs pathway induced by viral infections could represent the link between the upregulation of both the OAS gene family and the dsRNA virus network that we observed in our study.

## Supplementary Materials

Supplementary materials can be found at http://www.mdpi.com/1422-0067/19/9/2786/s1.

## Abbreviations

OAS2	2′-5′-oligoadenylate synthetase 2
OAS1	2′-5′-oligoadenylate synthetase 1
OAS3	2′-5′-oligoadenylate synthetase 3
OASL	2′-5′-oligoadenylate synthetase-like
dsRNA	double-stranded RNA
IPS1	also known as VISA: IFN-β-promoter stimulator 1
MDA5	melanoma differentiation associated protein 5
MS	multiple sclerosis
RIG-I	retinoic acid-inducible gene I
RA	rheumatoid arthritis
MBx	skeletal muscle biopsies
SLE	systemic lupus erythematosus
Sc	systemic sclerosis
TBK1/IKKE	TANK-binding kinase
TRIF	TLR adaptor molecule 1
TLR3	Toll-like receptor 3
IRF3	transcription factor IFN regulatory factor 3
TRAF3	tumor necrosis factor (TNF) receptor-associated factor 3
