# Supplementary material for: Expression of the OAS Gene Family Is Highly Modulated in Subjects Affected by Juvenile Dermatomyositis, Resembling an Immune Response to a dsRNA Virus Infection
Source: Int J Mol Sci. 2018 Sep 17;19(9):2786. doi: 10.3390/ijms19092786 (PMC6163680; doi:10.3390/ijms19092786)
Supplement: Supplementary file 1 [file ijms-19-02786-s001.zip › 1. Supplementary Figures.pdf]

Supplementary Figures and tables

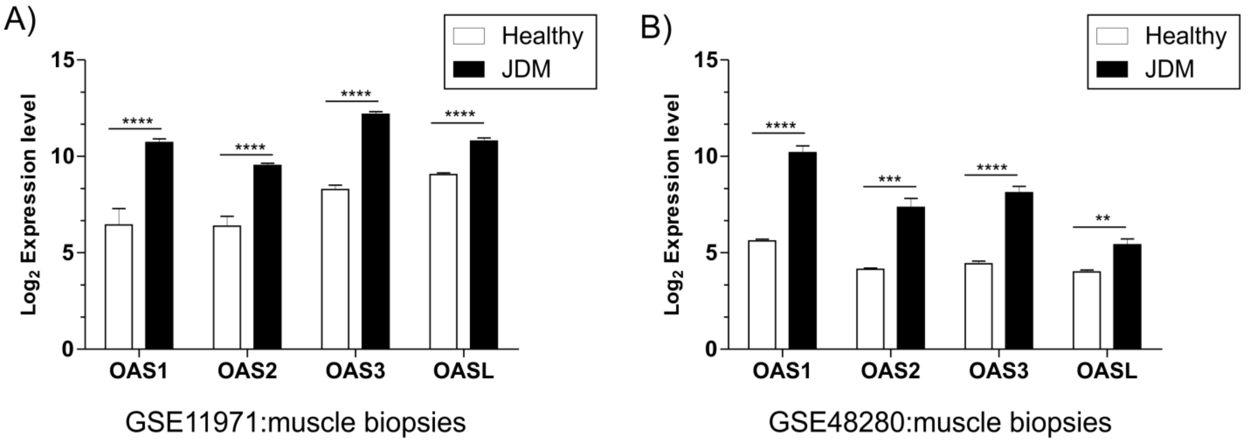

SF1: OASs genes expression level validation in GSE11971 and GSE48280

(A/B) OAS1, OAS2, OAS3 and OASL expression levels in muscles biopsy of JDM patients compared to healthy controls. Dataset accession number GSE11971 and GSE48280. Data are expressed as Log_2 intensity expression levels and presented as bar plots. P values <0.05 were considered to be statistically significant (*p<0.05; **p<0.005;***p<0.0005; ****p<0.00005).

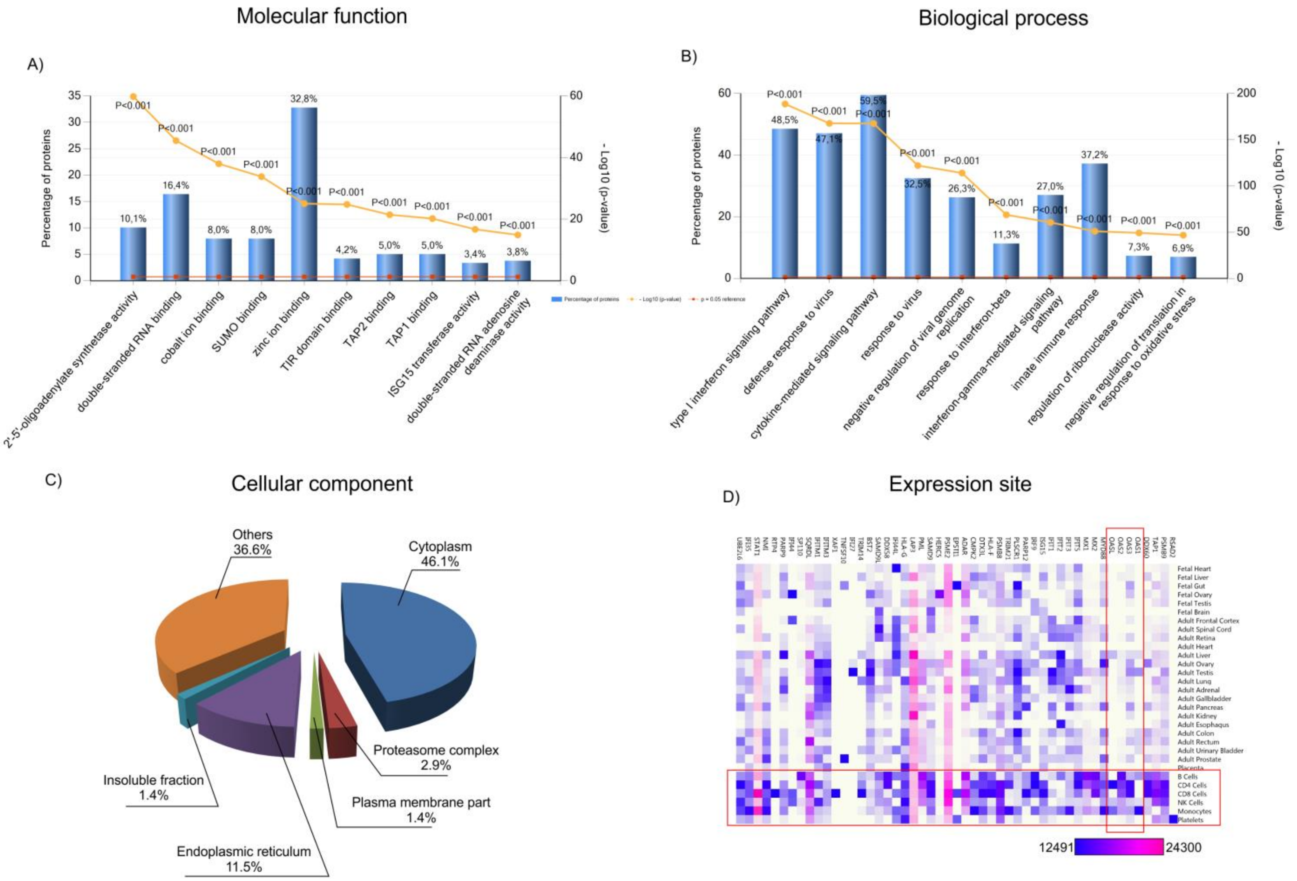

SF2: FunRich analysis

(A) The FunRich Molecular function analysis of 53 query genes upregulated in both the OASs gene network and JDM showed that 32.8% of genes have a zinc ion binding, 16.4% dsRNA binding and 10.1% 2’-5’-oligoadenylate synthetase (OAS) activity ($p<0.001$). (B) As for the Biological process analysis, the FunRich showed the following results: 59.9% cytokine signaling, 48.5% Type I interferon signaling, 47.1% defense response to virus, 37.2% innate immune response and 26.3% negative regulation of viral genome replication ($p<0.001$). (C) The analysis of Cellular component showed a prevalence of network genes in the cytoplasm (46.1%) (D) The Expression site heatmap showed a pleiotropic expression of OASs genes, in CD4, CD8, and natural killer cells and high expression in monocytes.

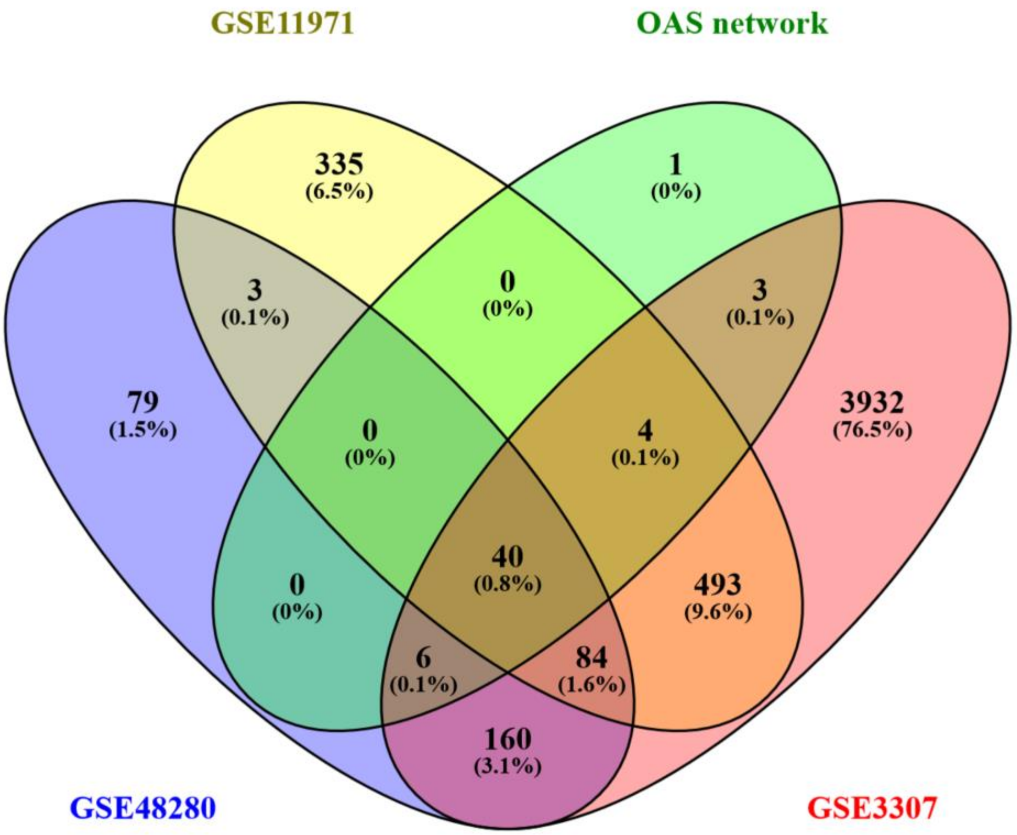

SF3: Venn Diagram analysis

Venn Diagrams analysis for significantly upregulated genes in three different microarray dataset (GSE3307, GSE11971 and GSE48280) vs OASs gene network obtained by the GIANT analysis. The analysis showed that 40 genes (74%) were in common to the JDM group vs. healthy control upregulated genes ($p<0.0001$ by Chi Square with Yates' correction).

Significant upregulated genes in GSE3307

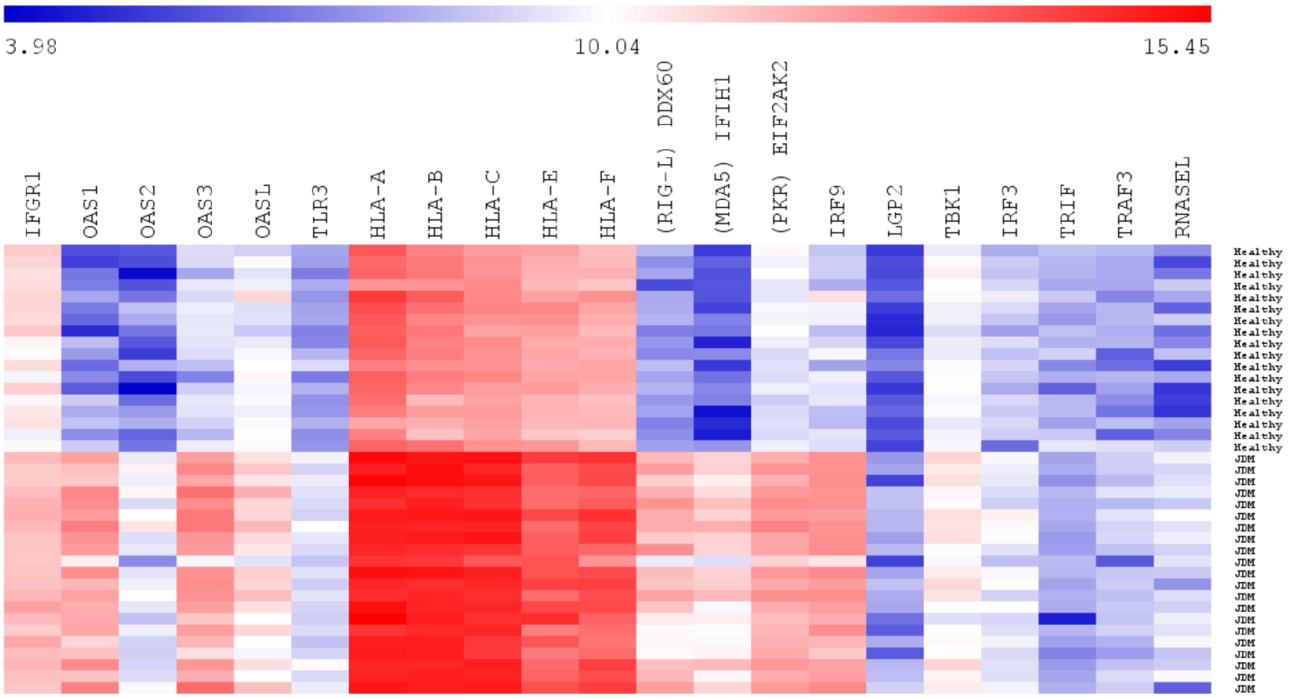

genes induced during dsRNA virus infection

SF4: Heatmap of significantly upregulated genes during dsRNA viral infections

The MultiExperiment Viewer (MeV) software was used to draw the Heatmap. Gene expression values are color-coded from bright red (highly upregulated) to dark blue (highly downregulated).

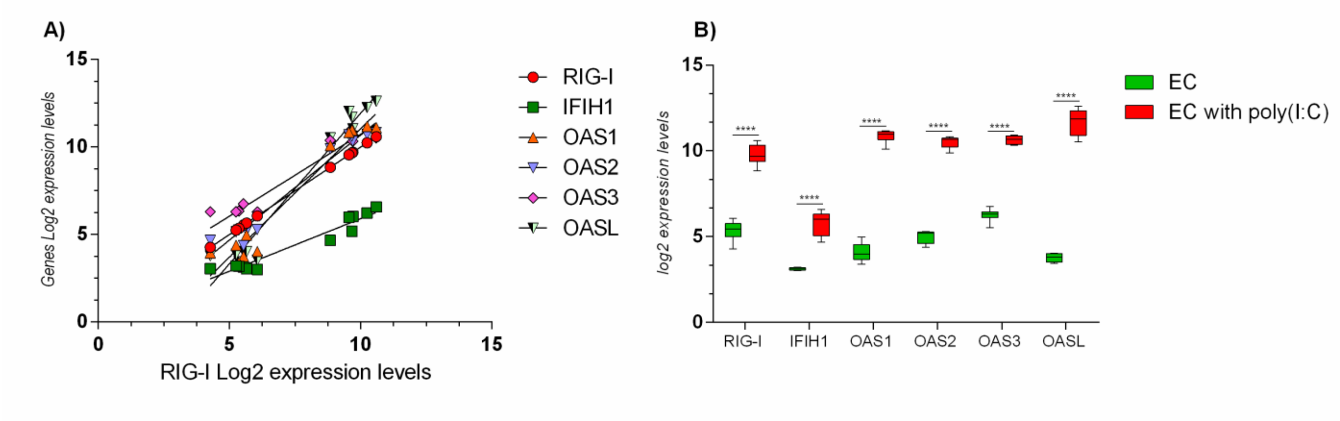

SF5: OASs genes family analysis in GSE51392

OASs genes family were positively correlated to RIG-1 and IFIH1 (A). All genes were significant upregulated in epithelial cells treated with poly(I:C) (20µg/ml for 24hrs). Data are expressed as Log_2 intensity expression levels and presented as bar plots. P values <0.05 were considered to be statistically significant (*p<0.05; **p<0.005;***p<0.0005; ****p<0.00005).

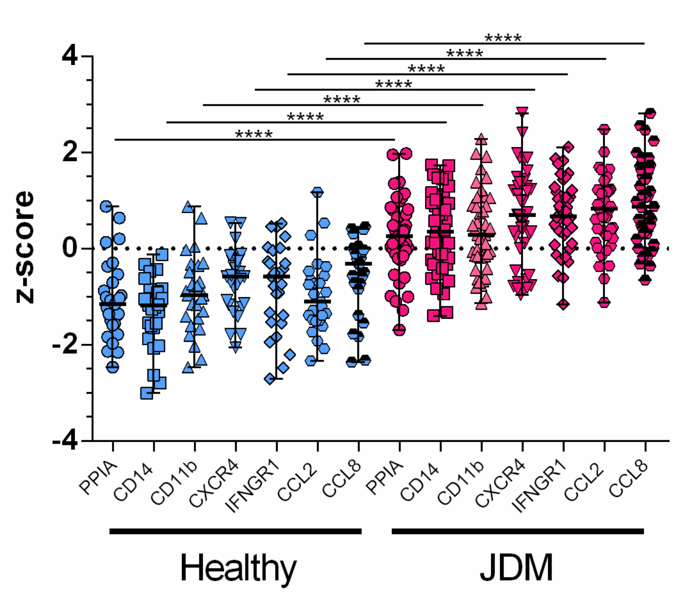

SF6: Expression analysis of necrotic marker (PPIA), monocyte markers (CD14, CD11b and CXCR4), monocyte infiltration markers (CCL2 and CCL8) and IFN gamma receptor (IFNGR1).

We analyzed the necrotic marker Cyclophilin a (PPIA), the monocyte markers CD14, CD11b and CXCR4, the monocyte infiltration markers CCL2 and CCL8 in the dataset used for this study. In order to improve the significantly statistic results, we performed a z-score analysis of the tree data set includes in our study. We showed that PPIa was significantly upregulated in JDM patients compared to the matched healthy subjects. As regards the monocyte markers, we analyzed the expression levels of CD14, CD11b, and CXCR4. We showed that all genes were significantly upregulated in JDM patients compared to healthy subjects. Also, the expression levels were positively correlated with OAS2, OAS3, and OASL (sTable 1A). To verify the hypothesis of monocytes infiltration in JDM muscle biopsies patients, we performed an expression analysis of CCL2 and CCL8 to two of the main chemokines for monocytes. The analysis showed that the two genes were significantly upregulated in patients with JDM and their levels correlated positively with both the OASs gene family and monocytes markers (sTable 1). Furthermore, we analyzed the expression levels of IFNG and its receptor (IFNGR1) in order to verify if this molecule plays a role in JDM. The analysis showed that only the IFNGR1 was significantly modulated. Altogether, these results show that the family of OASs genes is correlated with the

marker genes of the monocyte cells, with the main chemokines called in question during monocyte infiltration and with the interferon-gamma receptor. Data are expressed as Log_2 intensity expression levels and presented as dot plots. P values <0.05 were considered to be statistically significant (*p<0.05; **p<0.005;***p<0.0005; ****p<0.00005).

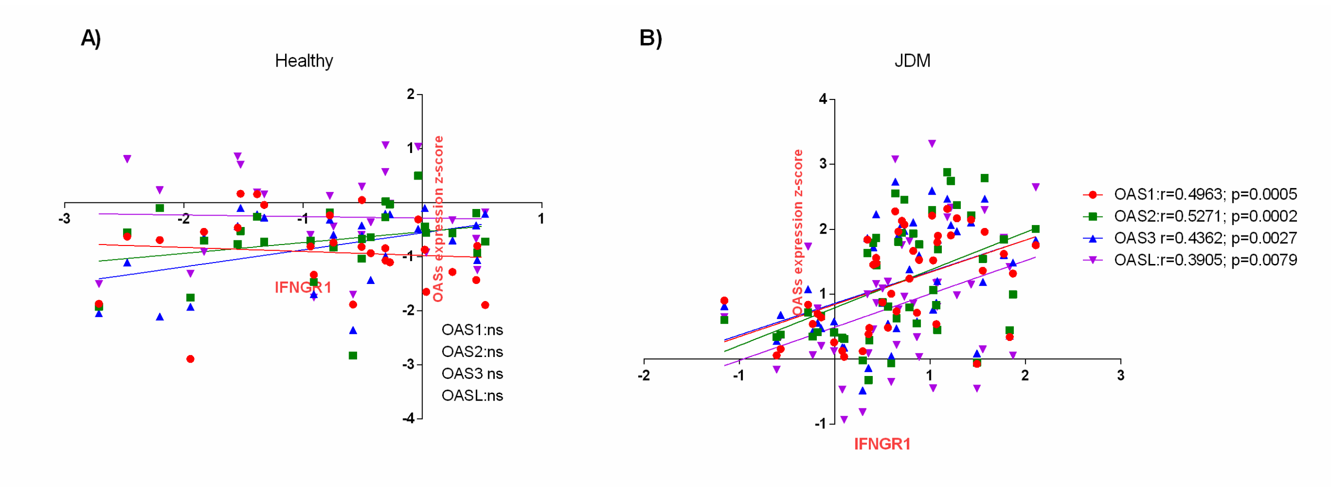

SF7: Correlation analysis of IFNGR1 with OASs genes family

The correlation analysis reviled that IFNGR1 was significantly correlated with the necrotic marker (PPIA), the monocyte markers (CXCR4), monocytes infiltration markers (CCL2 and CCL8) and the OASs genes family expression. Data are expressed as Log_2 intensity expression levels and presented as dot plots. P values <0.05 were considered to be statistically significant (*p<0.05; **p<0.005;***p<0.0005; ****p<0.00005).

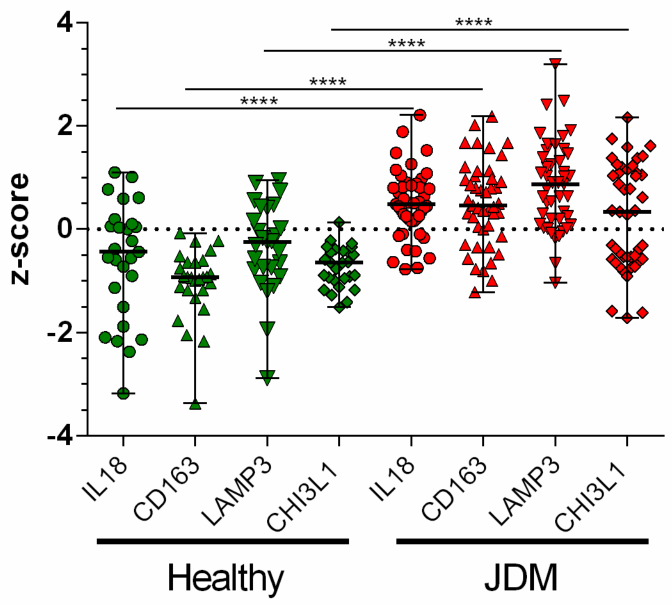

SF8: Expression analysis of activated innate immunity markers

We decided to investigate whether there was a correlation between the expression of the main markers of activated innate immunity in JDM muscular biopsies and the OASs genes family expression levels. In order to verify the link to OASs genes and innate immune cell activation, we have decided to analyze the expression levels of IL18, CD163, LAMP3, and CHI3L1. The interferon-gamma inducing factor 18 (also known Interleukin-18, IL18) is a pro-inflammatory cytokine produced by activated monocyte/macrophages. Induces the differentiation of CD4 + T lymphocytes into TH1 lymphocytes and the production of IFN-γ in T lymphocytes. In our analyses, we showed that the expression levels of IL18 was significantly expressed in JDM muscles biopsies and it levels positively correlate with OAS1, OAS2, and OAS3 (sTable 1B). The cluster of Differentiation 163 (CD163), is a marker of cells from the monocyte/macrophage lineage. Is highly expressed on the surface of activated macrophages. Serum sCD163 might be a potential biomarker for predicting the severity and prognosis of polymyositis and dermatomyositis. The analysis of CD163 in the dataset selected for our study showed a significant upregulation in JDM biopsies and a significantly positive correlation with OAS2, OAS3, and OASL (sTable 1B). The Lysosome-associated membrane glycoprotein 3 (also called DC-LAMP or LAMP3) is a marker of activated Dendritic cells. There is evidence that in skin from patients with juvenile DM, there is an increased number of infiltrated dendritic cells. The gene expression levels of LAMP3 in muscles biopsies of JDM patients compared with healthy subjects showed a significant increase. Furthermore, the expression levels were significantly correlated with all genes belonging to the OASs

family(sTable 1B). There is a large bibliography that identifies the CHI3L1 expression levels as an activation marker of innate immunity. In our analysis, we showed increased expression levels in JDM muscles biopsies compared to the healthy subjects. Also, the expression levels were significantly correlated with OAS2, OAS3, and OASL(sTable 1B). All this finding would seem to confirm an activation of innate immunity in the biopsies of patients with JDM and a possible correlation with the OAS family genes. Data are expressed as Log_2 intensity expression levels and presented as dot plots. P values <0.05 were considered to be statistically significant (*p<0.05; **p<0.005;***p<0.0005; ****p<0.00005).

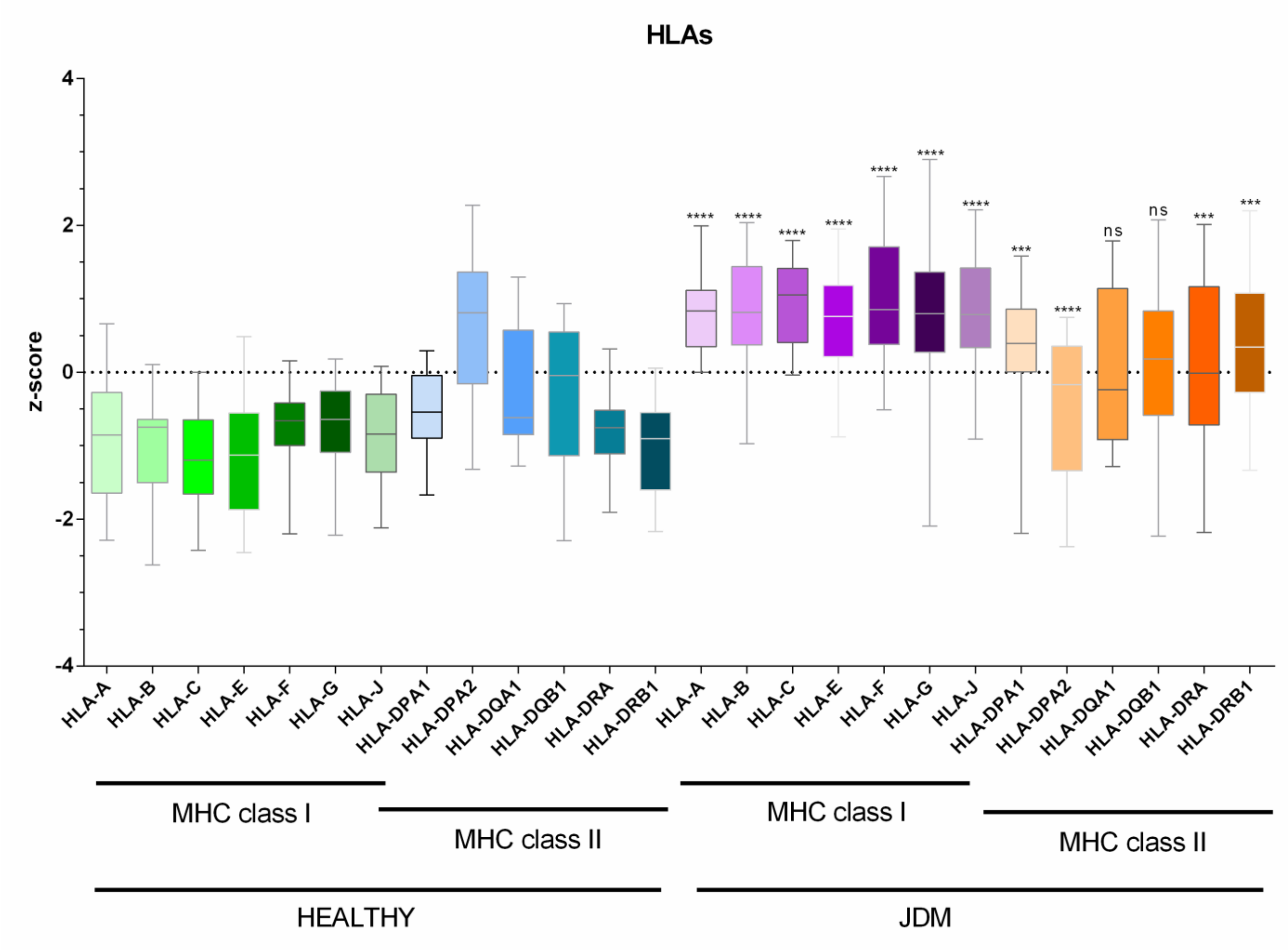

SF9: Expression analysis of MHC class I/II in JDM muscles biopsies

We performed the expression and correlation analysis between the OAS genes levels and the MHC class I /II. In the last years the role played by the MHC class I/II in JDM was largely studies. In our analysis, we showed that the expression levels of MHC class I/II in muscle biopsies of patients with JDM were significantly upregulated. The MHC class I levels were more expressed than MHC class II. Furthermore, the MHC class I expression levels were significantly correlated with the OAS genes expression. No correlation was observed between the OAS genes and the MHC class II. These results could be explained by the key role played by OASs genes and CD8 cytotoxic T lymphocyte during the viral infection (sTable 1C). Data are expressed as Log_2 intensity expression levels and presented as scatter plots. P values <0.05 were considered to be statistically significant (*p<0.05; **p<0.005;***p<0.0005; ****p<0.00005).

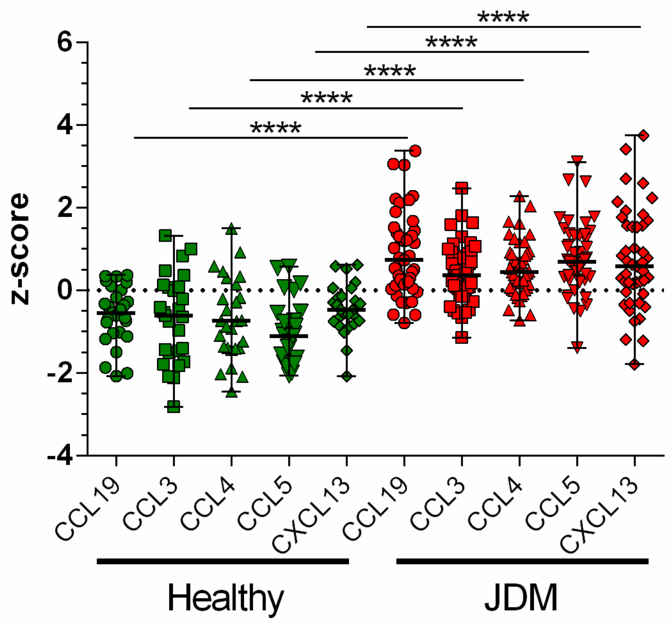

SF10: Expression analysis of adaptive immune response markers.

The principal chemokine involved in recruitment and activation of T and B lymphocytes are CCL19, CCL3, CCL4, CCL5, and CXCL13. The expression analysis of these chemokines in our datasets showed significant increases in JDM patients compared to healthy subjects. The correlation analysis between OAS genes expression levels and CCL19 showed significant results. As regard CCL4, CCL5 and CXCL13 we observed a significantly correlation with OAS1, OAS2, and OAS3. The CCL3 was only positively correlated with OASL (sTable 1D). These results could explain a possible recruitment and subsequent activation of adaptive immunity in muscular biopsies of patients affected by JDM. Data are expressed as Log_2 intensity expression levels and presented as dot plots. P values <0.05 were considered to be statistically significant (*p<0.05; **p<0.005;***p<0.0005; ****p<0.00005).
